# Supplementary material for: Low complexity domains of the nucleocapsid protein of SARS-CoV-2 form amyloid fibrils
Source: Nat Commun. 2023 Apr 25;14:2379. doi: 10.1038/s41467-023-37865-3 (PMC10127185; doi:10.1038/s41467-023-37865-3)
Supplement: Supplementary file 1 — Supplementary Information [file 41467_2023_37865_MOESM1_ESM.pdf]

Low Complexity Domains of the Nucleocapsid Protein of SARS-CoV-2 Form Amyloid Fibrils

Einav Tayeb-Fligelman[1,2,3,4], Jeannette T. Bowler[1,2,3,4], Christen E. Tai[1,2], Michael R. Sawaya[1,2,3,4,5], Yi Xiao Jiang[1,2,3,4], Gustavo Garcia Jr[6], Sarah L. Griner[1,2,3,4], Xinyi Cheng[1,2,3,4], Lukasz Salwinski[1,2,5], Liisa Lutter[1,2,3,4], Paul M. Seidler[1,2,†], Jiahui Lu[1,2,3,4], Gregory M. Rosenberg[1,2,3,4], Ke Hou[1,2,3,4], Romany Abskharon[1,2,3,4], Hope Pan[1,2,3,4], Chih-Te Zee[3], David R. Boyer[1,2,3,4], Yan Li[1,2], Daniel H. Anderson[1,2,3,4], Kevin A. Murray[1,2,3,4], Genesis Falcon[5], Duilio Cascio[5], Lorena Saelices[1,2 ††], Robert Damoiseaux[6,7,8,9,10], Vaithilingaraja Arumugaswami[6,8,9], Feng Guo[1,2,10], David S. Eisenberg[1,2,3,4,5,8*]

[1]Department of Biological Chemistry, UCLA, Los Angeles, CA 90095, USA. [2]Molecular Biology Institute, UCLA, Los Angeles, CA 90095, USA. [3]Department of Chemistry and Biochemistry, UCLA, Los Angeles, CA 90095, USA. [4]Howard Hughes Medical Institute, Los Angeles, CA 90095, USA. [5]UCLA-DOE Institute of Genomics and Proteomics, UCLA, Los Angeles, CA 90095, USA. [6]Department of Molecular and Medical Pharmacology, UCLA, Los Angeles, CA 90095, USA. [7]Department of Bioengineering, UCLA, Los Angeles, CA 90095, USA. [8]California NanoSystems Institute, UCLA, Los Angeles, CA 90095, USA. [9]Eli and Edythe Broad Center of Regenerative Medicine and Stem Cell Research, UCLA, Los Angeles, CA 90095, USA. [10]Jonsson Comprehensive Cancer Center, UCLA, Los Angeles, CA 90095, USA.

PRESENT ADDRESSES:

† Department of Pharmacology and Pharmaceutical Sciences, University of Southern California School of Pharmacy, Los Angeles, CA 90089-9121, USA.

†† Center for Alzheimer's and Neurodegenerative Diseases, Department of Biophysics, Peter O'Donnell Jr. Brain Institute, University of Texas Southwestern Medical Center, Dallas, TX 75390, USA.

*Email: david@mbi.ucla.edu.

KEYWORDS: SARS-CoV-2, COVID-19, amyloid, antiviral, low-complexity domain (LCD), phase separation (PS).

SUPPLEMENTRY RESULTS AND DISCUSSION

NCAP'S PHASE SEPARATION DROPLETS ARE POSITIVE TO THIOFLAVIN-S AND CONTAIN FIBROUS AGGREGATES

NCAP undergoes phase separation (PS) with hairpin-Site2 (S2hp) vRNA (in 40:1 NCAP: vRNA molar ratio) and partitions with the fluorescent amyloid-dye Thioflavin-S (ThS) in the presence and absence of the PS enhancing $ZnCl_2$[1] (Supplementary Figure 2. a). When incubated in PBS for 1 day, NCAP samples without vRNA form only small, weakly ThS fluorescent specks. More brightly fluorescent, small, irregular structures appear in those samples on day 6 of incubation (Supplementary Figure 2. a, left middle columns). In the presence of vRNA, with or without $ZnCl_2$, NCAP rapidly forms large spherical droplets with weak ThS-fluorescence (Supplementary Figure 2. a, right columns), and small, strongly ThS- fluorescent structures were observed to decorate these rather weakly fluorescent PS droplets. On day 6 of incubation, the droplets become brighter, larger, and less spherical, and are decorated by multiple ThS- dense structures (Supplementary Figure 2. a).

Using transmission electron microscopy (TEM) to visualize these samples (Supplementary Figure 2. b), we observed fibrillar assemblies in all NCAP containing samples after 6 days of incubation. When incubated alone, NCAP produced some elongated fibrils of several micrometers in length (Supplementary Figure 2. b. ii.), as well as much shorter ones of less than 200 nm. The NCAP+ $ZnCl_2$ sample revealed more abundant, gel-like fibrils (Supplementary Figure 2. b. iii.). In the NCAP+ S2hp sample, multiple heavily stained fibrils were coated with aggregated material (Supplementary Figure 2. b. iv.). The origin of fibril coating is unclear, but it is possible that it is the vRNA itself, or amorphous NCAP aggregates. In the NCAP sample containing S2hp and $ZnCl_2$, both coated (as in Supplementary Figure 2. b. iv.) and bare fibrils (Supplementary Figure 2. b. v.) were found. We also detected some sparse fibrils on grids of a more concentrated NCAP sample incubated with and without S2hp vRNA at 4:1 NCAP: vRNA molar ratio in PBS for 2 weeks (Supplementary Figure 2. c). Fibrils formed with the vRNA were morphologically different than those formed in its absence. The most abundant NCAP fibrils were, however, detected in a sample of NCAP incubated for 3 days with $ZnCl_2$ at low ionic strength conditions (2 mM Tris pH 8.0, 30 mM NaCl) (Supplementary Figure 2. d). The aggregation of NCAP, especially in the presence of S2hp vRNA, was also verified by following increase in turbidity and Thioflavin-T (ThT) signal over time (Supplementary Figure 2. e-f). Overall, these results suggest that our recombinant NCAP protein is forming ThS positive PS droplets and is capable of adopting fibrillar morphologies under various conditions in vitro.

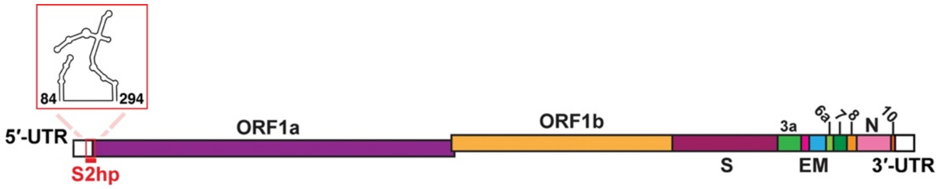

Supplementary Figure 1. **schematic representation of SARS-CoV-2 genome and the location of hairpin-Site2 (S2hp) vRNA used in this study.** The misalignment between ORF1a and ORF1b denotes a frameshift site.

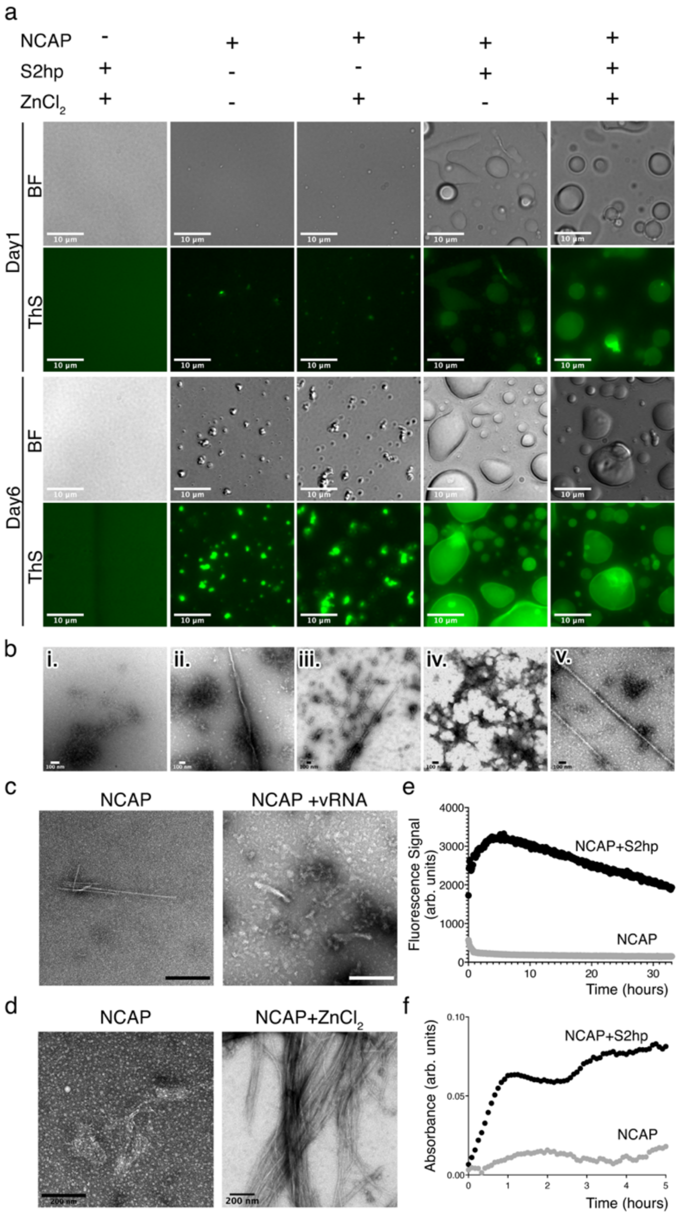

Supplementary Figure 2. **NCAP forms ThS positive droplets as well as fibrils and ThT positive species. a** Brightfield (BF) and fluorescence microscopy images of 30 µM Nucleocapsid protein (NCAP) samples incubated with Thioflavin-S (ThS; green) with and without 0.75 µM of hairpin-Site2 (S2hp) vRNA (40:1 NCAP: RNA molar ratio) and 20 µM $ZnCl_2$. The fluorescence intensity of PS droplets formed in the presence of both S2hp and $ZnCl_2$ at day 6 was high relative to the other conditions, and this image was adjusted separately to reduce brightness for clarity of features presented. **b** Negative stain transmission electron microscopy (TEM) micrographs of NCAP samples shown in a (in the same order) from day 6 of incubation. **c** TEM micrographs of 100 µM of NCAP incubated in PBS for 2 weeks with and without 25 µM of S2hp vRNA (4:1 NCAP: RNA molar ratio). **d** TEM micrographs of 50 µM NCAP incubated for 3 days in 2 mM Tris pH 8.0, 30 mM NaCl with 0 or 20 µM $ZnCl_2$. **e-f** Thioflavin-T (ThT) (**e**) and turbidity (**f**) measurements of 30 µM NCAP in the presence (black) and absence (gray) of 7.5 µM S2hp vRNA (4:1 NCAP: RNA molar ratio). Source data for panels e-f are provided as a Source Data file.

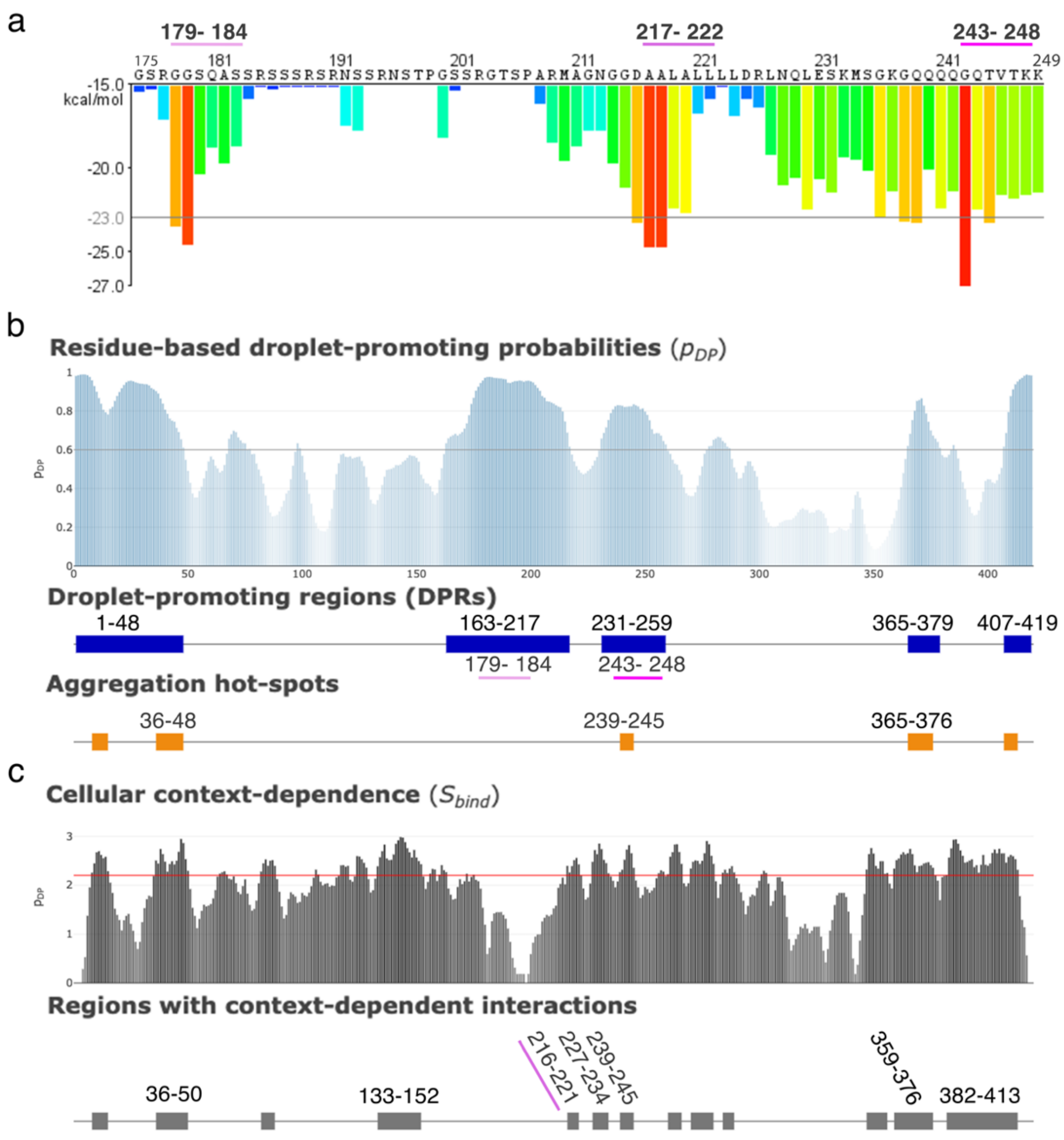

Supplementary Figure 3. **Computational prediction of PS and amyloidogenic sequences in NCAP**. **a** ZipperDB[2] [https://services.mbi.ucla.edu/zipperdb/] prediction of steric-zipper forming segments within the central LCD of NCAP. Three main six residue segments were identified as having high propensity to form amyloid. Those sequences are $_{179}$GSQASS$_{184}$, $_{217}$AALALL$_{222}$, and $_{243}$GQTVTK$_{248}$. The LCD sequence and residue positions are shown on the X-axis. The Y-axis shows gain of energy upon steric zipper formation. An energy threshold of -23 kcal/mol for steric-zipper formation is marked as a horizontal gray line. The bars represent six residue segments starting at the indicated position in the sequence. Orange-red segments are predicted to form fibrils. **b** Fuzdrop[3] [https://fuzdrop.bio.unipd.it/predictor] prediction of droplet promoting segments and aggregation hot-spots. Droplet promoting segments are labeled with blue boxes and residue numbers are shown on the top. The steric-zipper forming segments $_{179}$GSQASS$_{184}$ and $_{243}$GQTVTK$_{248}$ (marked with pink underlines and numbered on the plot) and part of the C-terminal LCD (residues 361-379) are also predicted to participate in droplet promoting interactions.

Aggregation hot-spots are marked with orange boxes and the $_{243}$GQTVTK$_{248}$ segment and the C-terminal LCD (residues 361-379) are partially included in those regions. **c** Fuzdrop prediction of regions prone to context dependent interactions, namely, regions that are capable of driving amyloid aggregation within PS droplets by switching between disordered and ordered interaction modes[3]. The majority of the $_{217}$AALALL$_{222}$ segment (part of the 216-221 segment that is depicted with a pink line) is predicted to participate in such context dependent interactions.

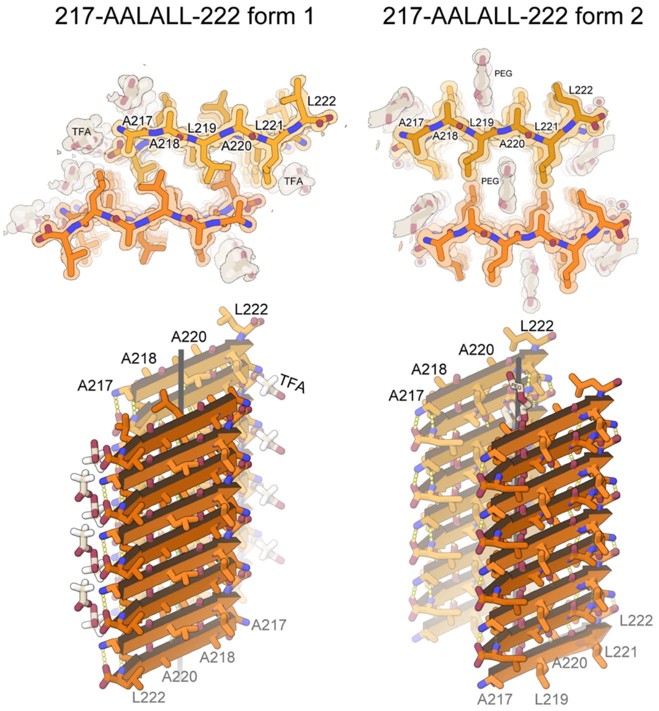

Supplementary Figure 4. (Expanded version of Figure 4) **Amyloid-like association of the NCAP segment AALALL revealed in two crystal forms.** The upper row shows the quality of the fit of each model to its corresponding simulated annealing composite omit maps[4]. The maps are contoured at the 1.0 sigma level. The structural features are well defined by the density. The view is directed down the fibril axis. Each chain shown here corresponds to one strand in a beta-sheet. Thousands of identical strands stack above and below the plane of the page making ~100 micron-long beta-sheets. The face of each beta-sheet of AALALL (PDB 7LTU [http://doi.org/10.2210/pdb7LTU/pdb] (form 1); PDB 7LUX [http://doi.org/10.2210/pdb7LUX/pdb] (form 2)) is symmetric with its back. The lower row shows 18 strands from each of the steric zippers at a view nearly perpendicular to the fibril axis. The AALALL zippers are antiparallel, in register sheets, mated with Class 7 zipper symmetry. Trifluoroacetic acid (TFA) appear bound to the AALALL- form 1 steric zipper, and Polyethylene glycol (PEG) to form 2. As the PEG is incorporated into the zipper interface in form 2, we postulate that this form is less likely to occur in vivo.

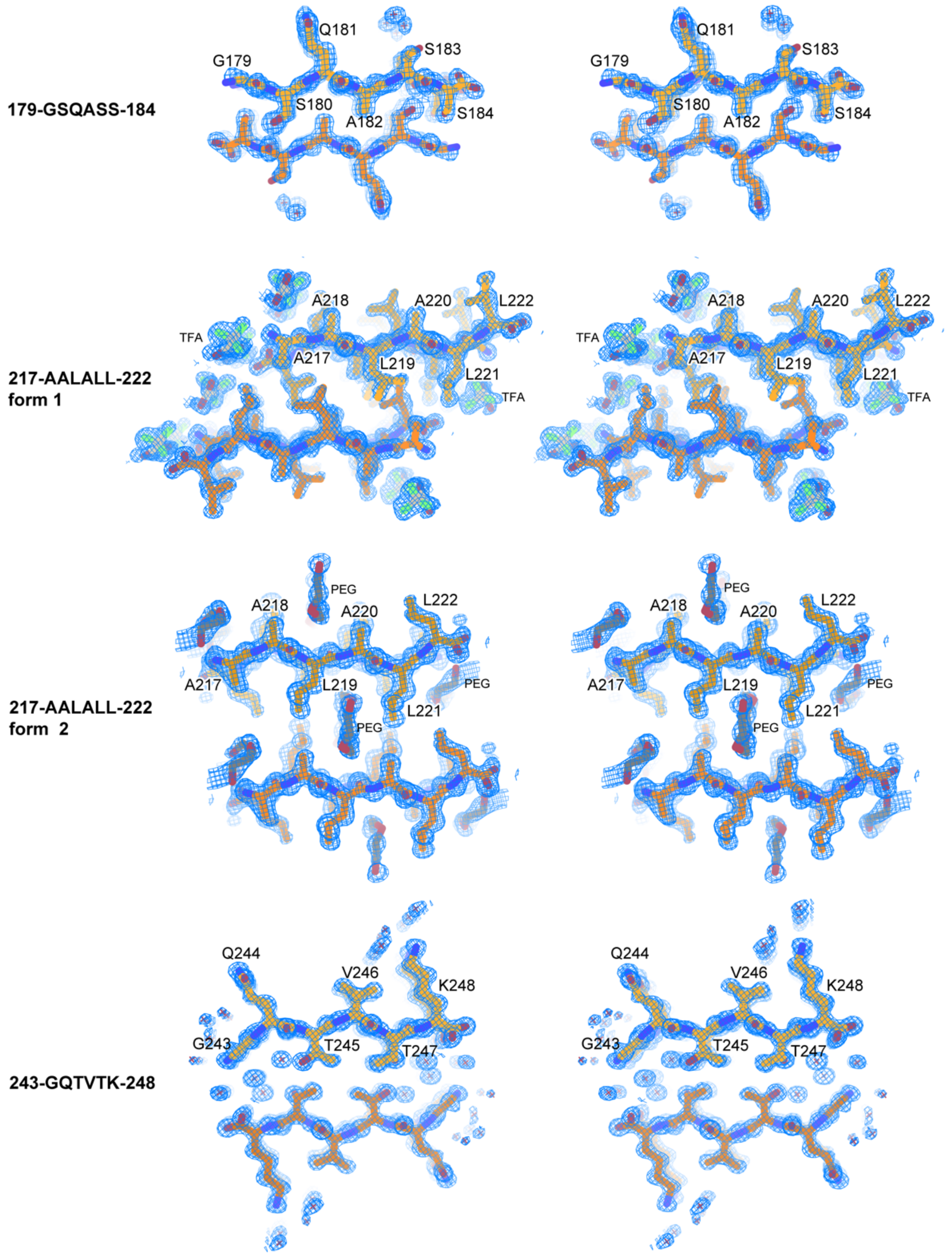

Supplementary Figure 5. **Stereo view illustration of the fit of atomic models into simulated annealing composite omit maps.** The maps are contoured at the 1.0 sigma level. The structural features are well defined by the density. The view is directed down the fibril axis.

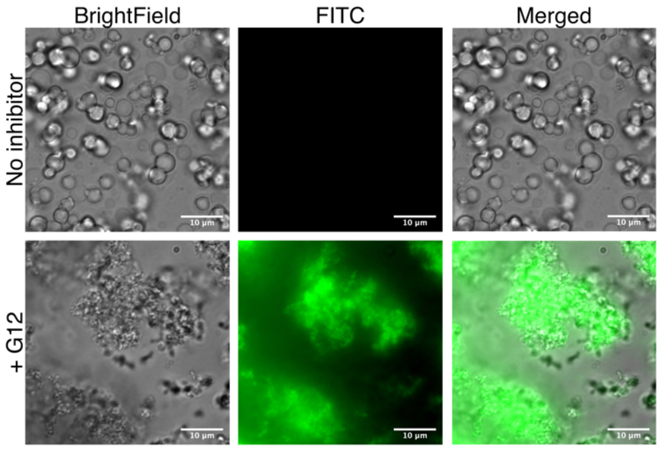

Supplementary Figure 6. **Localization of FITC-tagged G12 in NCAP assemblies.** PS droplets of NCAP were formed with S2hp vRNA and $ZnCl_2$. FITC-tagged G12 was mixed with untagged peptide at a 1: 9 ratio, and was then added to the samples at 1: 1 final molar ratio with NCAP. NCAP PS without G12 shows no FITC fluorescence. Addition of G12 induces formation of aggregates that contain the G12 peptide (green).

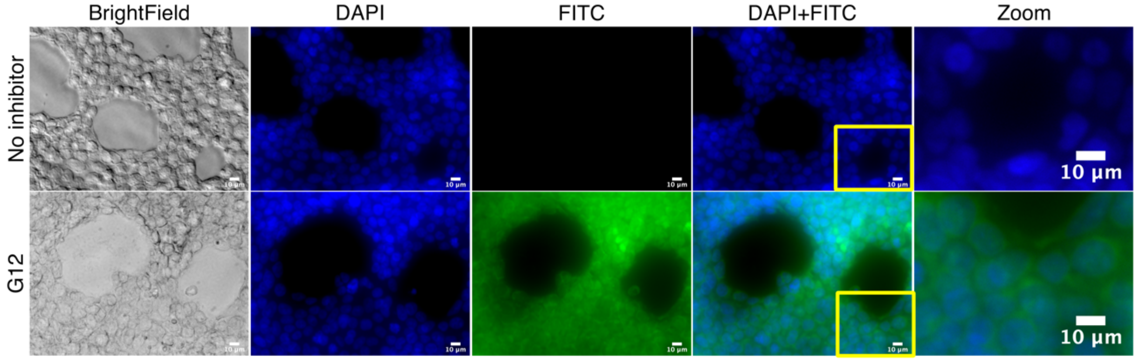

Supplementary Figure 7. **FITC-labeled G12 is diffused in transfected HEK293-ACE2 cells as visualized using fluorescence microscopy.** FITC-tagged G12 (green) was transfected into HEK293-ACE2 cells, which were then incubated for 24 hours at 37 ºC, 5% CO_2, then fixed and stained with DAPI (blue). The "zoom" inset on the right is an enlarged view of the yellow boxes in the composite DAPI+ FITC images. A 10 µm scale bar is shown on the right bottom side of each image.

Supplementary Figure 8. (Extended version of Figure 5. d). **A full dose-dependence analysis of G12 inhibition of SARS-CoV-2 infection in HEK293-ACE2 cells.** Cells were transfected with indicated concentrations of G12, infected 3-4 hours later with the virus and fixed at 24 hours post infection. The overall percentage of cells positive for infection in each sample were calculated via quantitative immunofluorescence labeling of the spike protein relative to the number of nuclei in each sample. The relative % infected was then achieved by normalizing the percentage of positive cells to the vehicle control (0 µM G12). Bars and dots indicate the mean and measured values of individual replicates, respectively.

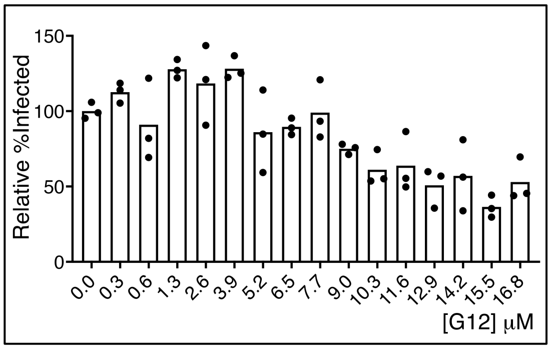

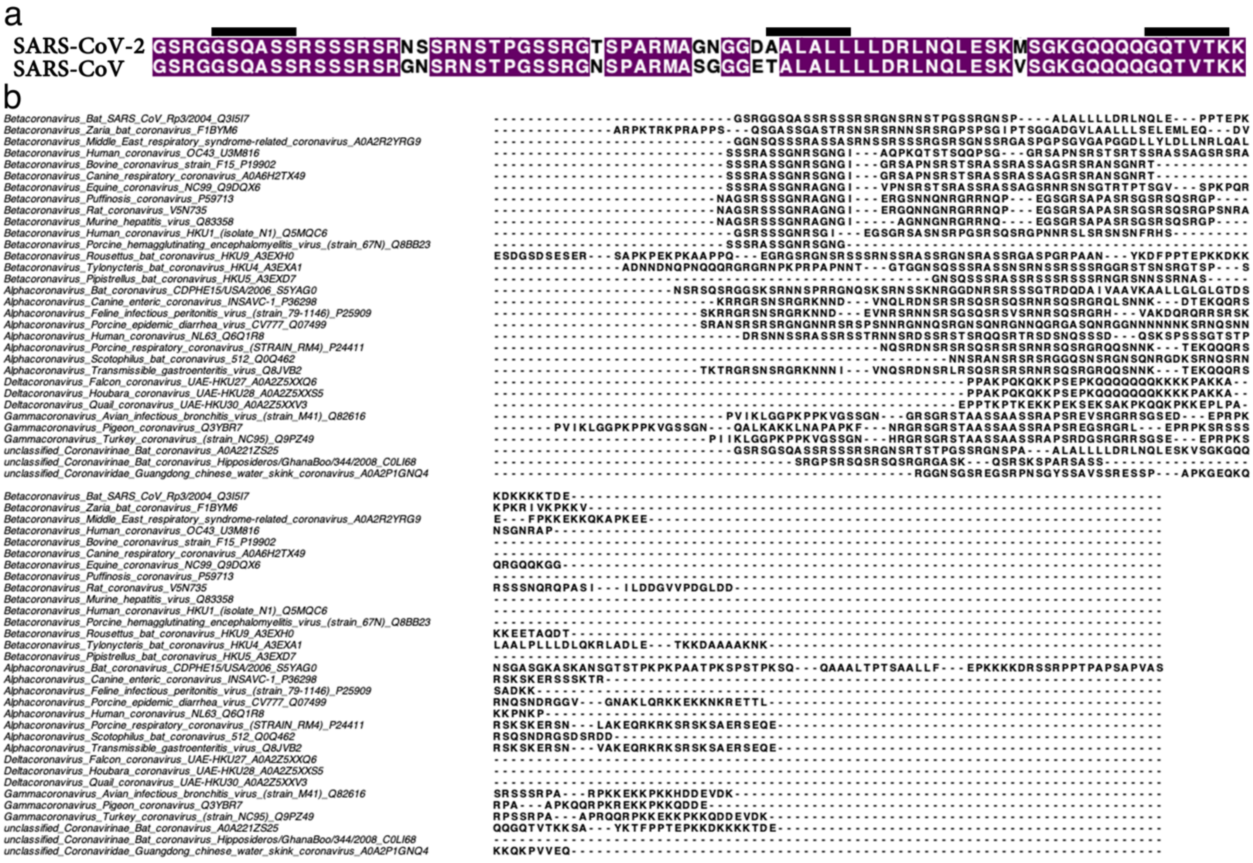

Supplementary Figure 9. **Conservation of NCAP's central LCD with that of SARS-CoV, and LCD identification in other coronaviral NCAPs.** **a** Sequence alignment of the central LCDs of the NCAPs of SARS-CoV-2 and SARS-CoV showing high level of conservation. Identical residues are colored white and highlighted in purple. The steric-zipper forming segments in NCAP of SARS-CoV-2 that were identified in this study are marked with an upper line. **b** Alignment of LCDs in NCAPs of representative coronaviruses. Sequences were aligned using ClustalW without allowing for gaps to be inserted into sequences. Here, gaps within a sequence represent an interrupting non-LCDs of at least 20% the length of the longest LCD of that protein. Some gaps were manually adjusted in size to achieve a better alignment. Sequence IDs contain the viral genus, name, and Uniprot ID of the NCAPs.

```
AAATTAATACGACTCACTATAGGGGAATTGTGAGCGGATAACAATTCCCCTCTAGAAATAATTTTGTTTAACTTTAAGAAGGAGATATACCATGGGCAGC
    T7

AGCCATCATCATCATCATCACATGTCGGACTCAGAAGTCAATCAAGAAGCTAAGCCAGAGGTCAAGCCAGAAGTCAAGCCTGAGACTCACATCAATTTAA

AGGTGTCCGATGGGTCTTCAGAGATCTTCTTCAAGATCAAAAAGACCACTCCTTTAAGAAGGCTGATGGAAGCGTTCGCTAAAAGACAGGGTAAGGAAAT

GGACTCCTTAAGATTCTTGTACGACGGTATTAGAATTCAAGCTGATCAGACCCCTGAAGATTTGGACATGGAGGATAACGATATTATTGAGGCTCACAGA
                                                 SEQ1F

GAACAGATTGGTGGTACCTCTGATAATGGACCCCAAAATCAGCGAAATGCACCCCGCATTACGTTTGGTGGACCCTCAGATTCAACTGGCAGTAACCAGA
             KpnI

ATGGAGAACGCAGTGGGGCGCGATCAAAACAACGTCGGCCCCAAGGTTTACCCAATAATACTGCGTCTTGGTTCACCGCTCTCACTCAACATGGCAAGGA

AGACCTTAAATTCCCTCGAGGACAAGGCGTTCCAATTAACACCAATAGCAGTCCAGATGACCAAATTGGCTACTACCGAAGAGCTACCAGACGAATTCGT

GGTGGTGACGGTAAAATGAAAGATCTCAGTCCAAGATGGTATTTCTACTACCTAGGAACTGGGCCAGAAGCTGGACTTCCCTATGGTGCTAACAAAGACG

GCATCATATGGGTTGCAACTGAGGGAGCCTTGAATACACCAAAAGATCACATTGGCACCCGCAATCCTGCTAACAATGCTGCAATCGTGCTACAACTTCC
                                                                                  SEQ2F

TCAAGGAACAACATTGCCAAAAGGCTTCTACGCAGAAGGGAGCAGAGGCGGCAGTCAAGCCTCTTCTCGTTCCTCATCACGTAGTCGCAACAGTTCAAGA

AATTCAACTCCAGGCAGCAGTAGGGGAACTTCTCCTGCTAGAATGGCTGGCAATGGCGGTGATGCTGCTCTTGCTTTGCTGCTGCTTGACAGATTGAACC

AGCTTGAGAGCAAAATGTCTGGTAAAGGCCAACAACAACAAGGCCAAACTGTCACTAAGAAATCTGCTGCTGAGGCTTCTAAGAAGCCTCGGCAAAAACG

TACTGCCACTAAAGCATACAATGTAACACAAGCTTTCGGCAGACGTGGTCCAGAACAAACCCAAGGAAATTTTGGGGACCAGGAACTAATCAGACAAGGA

ACTGATTACAAACATTGGCCGCAAATTGCACAATTTGCCCCCAGCGCTTCAGCGTTCTTCGGAATGTCGCGCATTGGCATGGAAGTCACACCTTCGGGAA

CGTGGTTGACCTACACAGGTGCCATCAAATTGGATGACAAAGATCCAAATTTCAAAGATCAAGTCATTTTGCTGAATAAGCATATTGACGCATACAAAAC

ATTCCCACCAACAGAGCCTAAAAAGGACAAAAAGAAGAAGGCTGATGAAACTCAAGCCTTACCGCAGAGACAGAAGAAACAGCAAACTGTGACTCTTCTT

CCTGCTGCAGATTTGGATGATTTCTCCAAACAATTGCAACAATCCATGAGCAGTGCTGACTCAACTCAGGCCtaatagGAGCTCCGTCGACAAGCTTGCG
                                                                         *  *  SacI

GCCGCACTCGAGCACCACCACCACCACCACTGAGATCCGGCTGCTAACAAAGCCCGAAAGGAAGCTGAGTTGGCTGCTGCCACCGCTGAGCAATAACTAG
                                                                                 T7term

CATAACCCCTTGGG
```

Supplementary Figure 10. **Fragment of the constructs that was used to express SUMO-tagged, full length NCAP protein.** NCAP coding sequence highlighted in yellow, stop codons are marked by stars, positions of start codon, KpnI and SacI subcloning sites and sequencing primers (T7, T7term, SEQ1F and SEQ2F) are underlined.

NCAP

```
ref/1-527        1 MGSSHHHHHHMSDSEVNQEAKPEVKPEVKPETHINLKVSDGSSEIFFKIKKTTPLRRLMEAFAKRQGKEMDSLRFLYDGI 80
SEQ1F/1-333        --------------------------------------------------------------------------------
SEQ2F/1-325        --------------------------------------------------------------------------------
T7term/1-330       --------------------------------------------------------------------------------

ref/1-527       81 RIQADQTPEDLDMEDNDIIEAHREQIGGTSDNGPQNQRNAPRITFGGPSDSTGSNQNGERSGARSKQRRPQGLPNNTASW 160
SEQ1F/1-333      1 ---------------XXXXXXSQXXIGGTSDNGPQNQRNAPRITFGGPSDSTGSNQNGERSGARSKQRRPQGLPNNTASW 65
SEQ2F/1-325        --------------------------------------------------------------------------------
T7term/1-330       --------------------------------------------------------------------------------

ref/1-527      161 FTALTQHGKEDLKFPRGQGVPINTNSSPDDQIGYYRRATRRIRGGDGKMKDLSPRWYFYYLGTGPEAGLPYGANKDGIIW 240
SEQ1F/1-333     66 FTALTQHGKEDLKFPRGQGVPINTNSSPDDQIGYYRRATRRIRGGDGKMKDLSPRWYFYYLGTGPEAGLPYGANKDGIIW 145
SEQ2F/1-325        --------------------------------------------------------------------------------
T7term/1-330     1 --------------------------------------------------------------------XXXXXXXRRHHM 12

ref/1-527      241 VATEGALNTPKDHIGTRNPANNAAIVLQLPQGTTLPKGFYAEGSRGGSQASSRSSSRSRNSSRNSTPGSSRGTSPARMAG 320
SEQ1F/1-333    146 VATEGALNTPKDHIGTRNPANNAAIVLQLPQGTTLPKGFYAEGSRGGSQASSRSSSRSRNSSRNSTPGSSRGTSPARMAG 225
SEQ2F/1-325      1 ---------------------------------XXXXXFYAEGSRGGSQASSRSSSRSRNSSRNSTPGSSRGTSPARMAG 47
T7term/1-330    13 VATEGALNXXKDHIGTRNPANNAAIVLQLPQGTTLPKGFYAEGSRGGSQASSRSSSRSRNSSRNSTPGSSRGTSPARMAG 92

ref/1-527      321 NGGDAALALLLLDRLNQLESKMSGKGQQQQGQTVTKKSAAEASKKPRQKRTATKAYNVTQAFGRRGPEQTQGNFGDQELI 400
SEQ1F/1-333    226 NGGDAALALLLLDRLNQLESKMSGKGQQQQGQTVTKKSAAEASKKPRQKRTATKAYNVTQAFGRRGPEQTQGNFGDQELI 305
SEQ2F/1-325     48 NGGDAALALLLLDRLNQLESKMSGKGQQQQGQTVTKKSAAEASKKPRQKRTATKAYNVTQAFGRRGPEQTQGNFGDQELI 127
T7term/1-330    93 NGGDAALALLLLDRLNQLESKMSGKGQQQQGQTVTKKSAAEASKKPRQKRTATKAYNVTQAFGRRGPEQTQGNFGDQELI 172

ref/1-527      401 RQGTDYKHWPQIAQFAPSASAFFGMSRIGMEVTPSGTWLTYTGAIKLDDKDPNFKDQVILLNKHIDAYKTFPPTEPKKDK 480
SEQ1F/1-333    306 RQGTDYKHWPQIAXLPPXXQRSXXCRAX                                                     333
SEQ2F/1-325    128 RQGTDYKHWPQIAQFAPSASAFFGMSRIGMEVTPSGTWLTYTGAIKLDDKDPNFKDQVILLNKHIDAYKTFPPTEPKKDK 207
T7term/1-330   173 RQGTDYKHWPQIAQFAPSASAFFGMSRIGMEVTPSGTWLTYTGAIKLDDKDPNFKDQVILLNKHIDAYKTFPPTEPKKDK 252

ref/1-527      481 KKKADETQALPQRQKKQQTVTLLPAADLDDFSKQLQQSMSSADSTQA                                  527
SEQ1F/1-333
SEQ2F/1-325    208 KKKADETQALPQRQKKQQTVTLLPAADLDDFSKQLQQSMSSADSTQA--ELRRQACGRTRAPPPPPLRSGC-QSPKGS-V 283
T7term/1-330   253 KKKADETQALPQRQKKQQTVTLLPAADLDDFSKQLQQSMSSADSTQA--ELRRQACGRTRAPPPPPLRSGC-QSPKXXXX 329

ref/1-527
SEQ1F/1-333
SEQ2F/1-325    284 GCCHR-AITSITPWGL-TGLXGFFAEXGTISXLXMXXAX-XXXRX                                    325
T7term/1-330   330 X
```

LCD

```
ref/1-203        1 MGSSHHHHHHMSDSEVNQEAKPEVKPEVKPETHINLKVSDGSSEIFFKIKKTTPLRRLMEAFAKRQGKEMDSLRFLYDGI 80
SEQ1F/1-137        --------------------------------------------------------------------------------
T7term/1-234     1 MGSSHHHHHHMSDSEVNQEAKPEVKPEVKPETHINLKVSDGSSEIFFKIKKTTPLRRLMEAFAKRQGKEMDSLRFLYDGI 80

ref/1-203       81 RIQADQTPEDLDMEDNDIIEAHREQIGGTFYAEGSRGGSQASSRSSSRSRNSSRNSTPGSSRGTSPARMAGNGGDAALAL 160
SEQ1F/1-137      1 ---------------XXXX-XSQXXIGGTFYAEGSRGGSQASSRSSSRSRNSSRNSTPGSSRGTSPARMAGNGGDAALAL 64
T7term/1-234    81 RIQADQTPEDLDMEDNDIIEAHREQIGGTFYAEGSRGGSQASSRSSSRSRNSSRNSTPGSSRGTSPARMAGNGGDAALAL 160

ref/1-203      161 LLLDRLNQLESKMSGKGQQQQGQTVTKKSAAEASKKPRQKR**--------------------------------      203
SEQ1F/1-137     65 LLLDRLNQLESKMSGKGQQQQGQTVTKKSAAEASKKPRQKR**ELRRQACGRTRAPPPPPLRSGC-QSPKGS-VG      137
T7term/1-234   161 LLLDRLNQLESKMSGKGQQQQGQTVTKKSAAEASKKPRQKR**ELRRQACGRTRAPPPPPLRSGC-QXXXXXXXX      234
```

DD-Cterm

```
ref/1-358        1 MGSSHHHHHHMSDSEVNQEAKPEVKPEVKPETHINLKVSDGSSEIFFKIKKTTPLRRLMEAFAKRQGKEMDSLRFLYDGI 80
SEQ1F/1-330        --------------------------------------------------------------------------------
T7term/1-332     1 ----------------------------------------------------------XKRSLXXQGXXMDSXXFXXDGI 22

ref/1-358       81 RIQADQTPEDLDMEDNDIIEAHREQIGGTFYAEGSRGGSQASSRSSSRSRNSSRNSTPGSSRGTSPARMAGNGGDAALAL 160
SEQ1F/1-330      1 ----------------XXXEAHREQIGGTFYAEGSRGGSQASSRSSSRSRNSSRNSTPGSSRGTSPARMAGNGGDAALAL 64
T7term/1-332    23 RIQADQTPEDLDMEXXDIIEAHREQIGGTFYAEGSRGGSQASSRSSSRSRNSSRNSTPGSSRGTSPARMAGNGGDAALAL 102

ref/1-358      161 LLLDRLNQLESKMSGKGQQQQGQTVTKKSAAEASKKPRQKRTATKAYNVTQAFGRRGPEQTQGNFGDQELIRQGTDYKHW 240
SEQ1F/1-330     65 LLLDRLNQLESKMSGKGQQQQGQTVTKKSAAEASKKPRQKRTATKAYNVTQAFGRRGPEQTQGNFGDQELIRQGTDYKHW 144
T7term/1-332   103 LLLDRLNQLESKMSGKGQQQQGQTVTKKSAAEASKKPRQKRTATKAYNVTQAFGRRGPEQTQGNFGDQELIRQGTDYKHW 182

ref/1-358      241 PQIAQFAPSASAFFGMSRIGMEVTPSGTWLTYTGAIKLDDKDPNFKDQVILLNKHIDAYKTFPPTEPKKDKKKKADETQA 320
SEQ1F/1-330    145 PQIAQFAPSASAFFGMSRIGMEVTPSGTWLTYTGAIKLDDKDPNFKDQVILLNKHIDAYKTFPPTEPKKDKKKKADETQA 224
T7term/1-332   183 PQIAQFAPSASAFFGMSRIGMEVTPSGTWLTYTGAIKLDDKDPNFKDQVILLNKHIDAYKTFPPTEPKKDKKKKADETQA 262

ref/1-358      321 LPQRQKKQQTVTLLPAADLDDFSKQLQQSMSSADSTQA                                           358
SEQ1F/1-330    225 LPQRQKKQQTVTLLPAADLDDFSKQLQQSMSSADSTXA**ELRRQACGRTRAPPPPPLRSGC-QSPKXKLSWLLPPLXNN 303
T7term/1-332   263 LPQRQKKQQTVTLLPAADLDDFSKQLQQSMSSADSTQA**ELRRQACGRTRAPPPPPLRSGC-QSPXXXXX          332

ref/1-358
SEQ1F/1-330    304 -HNPXGPLTGXXGXFFAEXXXLYPGXXX                                                     330
T7term/1-332
```

Supplementary Figure 11. **Aligned translations of Sanger sequencing reads that fully cover fragments of NCAP gene subcloned at KpnI/SacI sites.** Depending on construct, T7, T7term, SEQ1F and SEQ2F sequencing primers were used. Original ABI electropherogram files (.ab1) are available upon request.

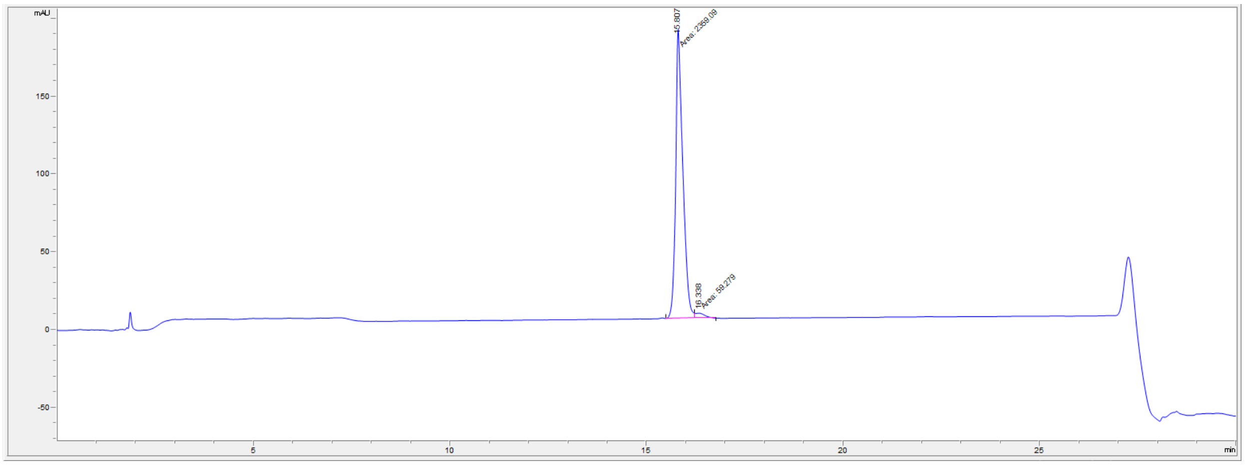

Supplementary Figure 12. **Analytical HPLC trace for purified AALALL.** The analytes are detected by their absorbance (y-axis, mAU) at 214 nm as they pass through the flow cell over time (x-axis, minutes). Peak areas were manually integrated. $\mathbf{t_R}$ **15.807**: 2359.1 mAU^2 (97.549%); $\mathbf{t_R}$ **16.338**: 59.3 mAU^2 (2.451%).

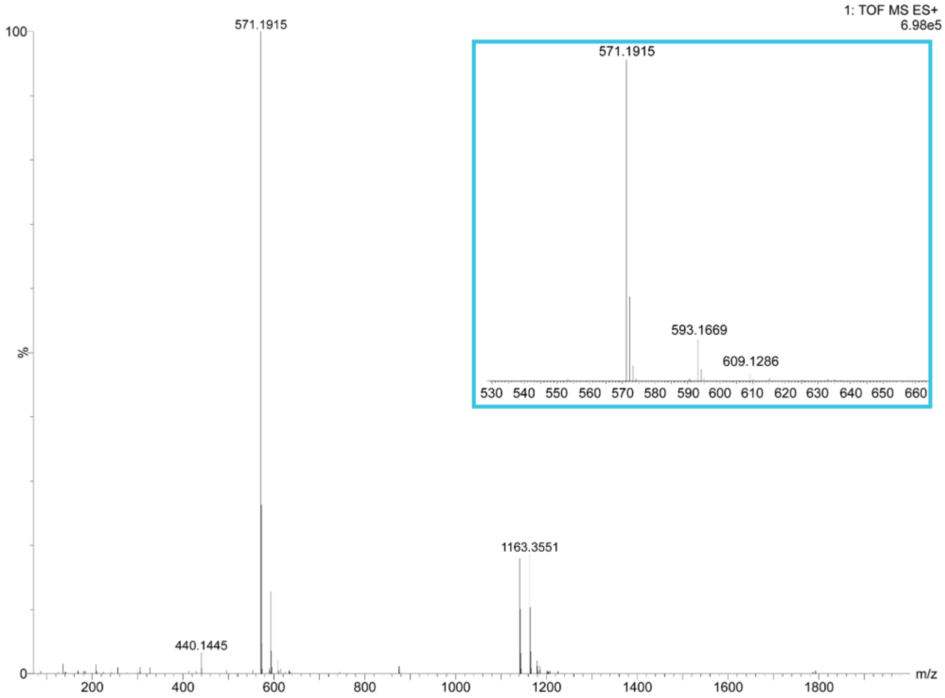

Supplementary Figure 13**. Broadband mass spectrum of purified AALALL collected by direct injection into a Waters LCT Premier Mass Spectrometer.** The scan range was 100-2000 (m/z), and the population of each ion is represented by relative abundance. The calculated monoisotopic mass for AALALL is 570.363 g/mol, m/z calculated: $[M+1H]^{1+}$ = 571.371; $[2M+1H]^{1+}$ = 1141.734. Observed: 571.191; 1141.383.

RNA name	Origin	RNA sequence
Site1 (S1)	5'-end gRNA [34-44]	5'-AACCAACUUUC-3'
Site1.5 (S1.5)	5'-end gRNA [60-83]	5'-UGUUCUCUAAACGAACUUUAAAAU-3'
Site2 (S2)	5'-end gRNA [128-149]	5'-UAUAAUUAAUAACUAAUUACUG-3'
Hairpin Site2 (S2hp)	5'-end gRNA [84-294]	5'-CTGTGTGGCTGTCACTCGGCTGCATGCTTAGTGCACTCACG CAGTATAATTAATAACTAATTACTGTCGTTGACAGGACACGAGT AACTCGTCTATCTTCTGCAGGCTGCTTACGGTTTCGTCCGTGTT GCAGCCGATCATCAGCACATCTAGGTTTCGTCCGGGTGTGACC GAAAGGTAAGATGGAGAGCCTTGTCCCTGGTTTCAACGA-3'
antisense siDGCR8-1[5]	human	5'-AUCACACUCUUGUCCGAUGUU-3'

Supplementary Table 1. **RNA sequences used in this work.** The RNA segments S1, S1.5, S2, and S2hp are derived from the genomic RNA (gRNA) sequence of SARS-CoV-2. Nucleotide sequence boundaries are given within square brackets.

Peptide Structure	Area buried in zipper interface per chain (Å2)	Shape complementarity	ΔG°/chain (kcal/mol)	ΔG°/residue (kcal/mol)
179-GSQASS-184	164	0.89	-0.9	-0.2
217-AALALL-222 form 1	73	0.81	-6.2	-1.0
217-AALALL-222 form 2	155	0.78	-6.5	-1.1
243-GQTVTK-248	102	0.39	-1.4	-0.2

Supplementary Table 2. **Steric zipper structural stability statistics.**

Inhibitor Name	Sequence	Target Structure	Design Approach	Rosetta Score[(a)]
G12	d-(rrffmvlm)	AALALL	Rosetta-based	-34.5 (fibril top)<br>-35.7 (fibril bottom)*

Supplementary Table 3. **Sequence and Rosetta scores of G12 binding to an AALALL fibril.**

(a) An arbitrary energetic score calculated for the binding of the designed peptide inhibitor to the fibril tip[6]. These scores were calculated without the terminal arginine chain that was added to increase the solubility of G12.

*The Rosetta score for the binding of additional AALALL strand for comparison is -30.78 (fibril top) and -34.57 (fibril bottom).

**Primer**	**Sequence**	**Constructs**	**Description**
NCAP-1	gatataccatgggcagcagccatcatcatc	NCAP	5' flanking (His-SUMO), NcoI
NCAP-2	tcgacggagctcctattaggcctgagttgagtcagcact	NCAP, DD-C_{term}	3' flanking, SacI
NCAP-4	tcgacggagctcctattaacgtttttgccgaggcttctt	LCD	3' flanking, SacI
NCAP-5	attggtggtacctctgataatggaccccaaaatcagcga	NCAP	SUMO/NCAP SOE (forward), KpnI
NCAP-6	gtccattatcagaggtaccaccaatctgttctctgtgagcctc	NCAP	SUMO/NCAP SOE (reverse), KpnI
NCAP-7	gccataggtaccttctacgcagaagggagcaga	LCD	5' flanking, KpnI
NCAP-8	tggtggtaccaagcctcggcaaaaacgtact	DD-C_{term}	5' flanking, KpnI

Supplementary Table 4. **PCR Primers.**

**AALALL QC Analysis**		
Time	[A]	[B]
(min)	(%)	(%)
0	90	10
5	80	20
25	60	40
26	0	100
30	0	100

Supplementary Table 5. **Gradient utilized for AALALL peptide purity analysis shown in Supplementary Figures 12 and 13.**

SUPPLEMENTARY REFERENCES:

1. Chen, H., Cui, Y., Han, X., Hu, W., Sun, M., Zhang, Y., Wang, P. H., Song, G., Chen, W. & Lou, J. Liquid–liquid phase separation by SARS-CoV-2 nucleocapsid protein and RNA. *Cell Res.* **30**, 1143–1145 (2020).
2. Goldschmidt, L., Teng, P. K., Riek, R. & Eisenberg, D. Identifying the amylome, proteins capable of forming amyloid-like fibrils. *Proc. Natl. Acad. Sci. U. S. A.* **107**, 3487–3492 (2010).
3. Hatos, A., Tosatto, S. C. E., Vendruscolo, M. & Fuxreiter, M. FuzDrop on AlphaFold: visualizing the sequence-dependent propensity of liquid-liquid phase separation and aggregation of proteins. *Nucleic Acids Res.* **50**, W337–W344 (2022).
4. Terwilliger, T. C., Grosse-Kunstleve, R. W., Afonine, P. V., Moriarty, N. W., Adams, P. D., Read, R. J., Zwart, P. H. & Hung, L. W. Iterative-build OMIT maps: Map improvement by iterative model building and refinement without model bias. *Acta Crystallogr. Sect. D Biol. Crystallogr.* **64**, 515–524 (2008).
5. Han, J., Lee, Y., Yeom, K. H., Kim, Y. K., Jin, H. & Kim, V. N. The Drosha-DGCR8 complex in primary microRNA processing. *Genes Dev.* **18**, 3016–3027 (2004).
6. Leaver-Fay, A., Tyka, M., Lewis, S. M., Lange, O. F., Thompson, J., Jacak, R., Kaufman, K., Renfrew, P. D., Smith, C. A., Sheffler, W., Davis, I. W., Cooper, S., Treuille, A., Mandell, D. J., Richter, F., Ban, Y. E. A., Fleishman, S. J., Corn, J. E., Kim, D. E., *et al.* Rosetta3: An object-oriented software suite for the simulation and design of macromolecules. *Methods Enzymol.* **487**, 545–574 (2011).
