# Supplementary material for: Low complexity domains of the nucleocapsid protein of SARS-CoV-2 form amyloid fibrils
Source: Nat Commun. 2023 Apr 25;14:2379. doi: 10.1038/s41467-023-37865-3 (PMC10127185; doi:10.1038/s41467-023-37865-3)
Supplement: Supplementary file 3 — Reporting Summary [file 41467_2023_37865_MOESM3_ESM.pdf]

nature portfolio

Corresponding author(s): David S. Eisenberg

Last updated by author(s): Mar 27, 2023

Reporting Summary

Nature Portfolio wishes to improve the reproducibility of the work that we publish. This form provides structure for consistency and transparency in reporting. For further information on Nature Portfolio policies, see our Editorial Policies and the Editorial Policy Checklist.

Statistics

For all statistical analyses, confirm that the following items are present in the figure legend, table legend, main text, or Methods section.

n/a	Confirmed	
☐	☒	The exact sample size (*n*) for each experimental group/condition, given as a discrete number and unit of measurement
☐	☒	A statement on whether measurements were taken from distinct samples or whether the same sample was measured repeatedly
☐	☒	The statistical test(s) used AND whether they are one- or two-sided<br>*Only common tests should be described solely by name; describe more complex techniques in the Methods section.*
☒	☐	A description of all covariates tested
☐	☒	A description of any assumptions or corrections, such as tests of normality and adjustment for multiple comparisons
☐	☒	A full description of the statistical parameters including central tendency (e.g. means) or other basic estimates (e.g. regression coefficient) AND variation (e.g. standard deviation) or associated estimates of uncertainty (e.g. confidence intervals)
☐	☒	For null hypothesis testing, the test statistic (e.g. *F*, *t*, *r*) with confidence intervals, effect sizes, degrees of freedom and *P* value noted<br>*Give P values as exact values whenever suitable.*
☒	☐	For Bayesian analysis, information on the choice of priors and Markov chain Monte Carlo settings
☒	☐	For hierarchical and complex designs, identification of the appropriate level for tests and full reporting of outcomes
☒	☐	Estimates of effect sizes (e.g. Cohen's *d*, Pearson's *r*), indicating how they were calculated

Our web collection on statistics for biologists contains articles on many of the points above.

Software and code

Policy information about availability of computer code

Data collection	Data collection and generation:<br>1. SEG- prediction of low complexity domains: http://rothlab.ucdavis.edu/genhelp/seg.html<br>2. ZipperDB- prediction of steric zippers: https://services.mbi.ucla.edu/zipperdb/<br>3. Fuzdrop- prediction of liquid-liquid phase separation, aggregation hot spots and context dependent interactions promoting segments: https://fuzdrop.bio.unipd.it/predictor<br>4. ZEISS ZEN 2 (blue addition)<br>5. Gatan DigitalMicrograph Suite v.1.8.5- for electron microscopy data collection<br>6. Rosetta3- design and modeling of peptide-based disruptors of NCAP's self-assembly.<br>7. BMG LABTECH- Omega s5.50 R4<br>8. SoftMax Pro v5.3<br>9. ImageXpress Micro Confocal High-Content Imaging System (Molecular Devices)
Data analysis	-BMG LABTECH- MARS 3.32 R5<br>-Image J (Fiji) v.1.53q for processing and image preparation of light, florescence and electron microscopy data<br>-MATLAB 9.13.0 (R2022b), which additionally use the Image Processing Toolbox 11.6 (R2022b) - quantification of light microscopy data<br>-Canny algorithm implemented in MATLAB 9.13.0 (R2022b), Image Processing Toolbox 11.6 (R2022b)<br>-Sobel approximation algorithm implemented in MATLAB 9.13.0 (R2022b), Image Processing Toolbox 11.6 (R2022b)

-GraphPad Prism 9- generation of plots and statistical analysis.
-Microsoft Excel version 16
-CCP4 suit v. 7.0.078 [https://www.ccp4.ac.uk/] including AREAIMOL and Sc for atomic structure determination of steric-zipper forming segments and for buried surface area and shape complementarity scoring of peptide-based designs.
-XDS Version Jan 31, 2020 BUILT=20200131 - Structure determination
-XSCALE Version Jan 31, 2020 BUILT=20200131 - Structure determination
-Phaser V. 2.8.3 - Structure determination
-PyMOL v. 2.5.2 for inspection of peptide-based designs and for figures of steric-zipper structures.
-UCSF Chimera v.1.15- For assigning Kyte and Doolittle hydrophobicity scores for residues in our steric-zipper structures and for respective figure rendering.
-MetaXpress version 6.1.0.2071, Multiwavelength Cell Scoring Application Module for analysis of antiviral activity of G12 in HEK293-ACE2 cells.
-ADXV v. 1.9.13- A program to display X-Ray diffraction images for fibril diffraction [https://www.scripps.edu/tainer/arvai/adxv.html]
- Adobe Illustrator 2003 v. 27.2- for figure preparation
-ShelxD 2013/2: for atomic structure determination [Sheldrick, G. M. Acta Crystallogr. Sect. D Biol. Crystallogr. 66, 479–485 (2010)]
-Refmac5 v.5.8.0267: for atomic structure refinement [Murshudov, G. N. et al. Acta Crystallogr. Sect. D Biol. Crystallogr. 67, 355–367 (2011)].
-Phenix v.1.18.2 release tag 3874: for generation of simulated annealing composite omit map [Afonine, P. V. et al. Acta Crystallogr. Sect. D Biol. Crystallogr. 68, 352–367 (2012)].
-Coot v.0.9.3: Model building of steric-zipper structures [Emsley, P., Lohkamp, B., Scott, W. G. & Cowtan, K. Acta Crystallogr. Sect. D Biol. Crystallogr. 66, 486–501 (2010)].
- Solvation free energy estimates were calculated using the software available here: doi:10.5281/zenodo.6321286. https://doi.org/10.5281/zenodo.6321286. Software version 7.2.
-JalView v. 2.11.1.2
-BioEdit v. 7.2.5
-ClustalW v. 2.1

For manuscripts utilizing custom algorithms or software that are central to the research but not yet described in published literature, software must be made available to editors and reviewers. We strongly encourage code deposition in a community repository (e.g. GitHub). See the Nature Portfolio guidelines for submitting code & software for further information.

Data

Policy information about availability of data

All manuscripts must include a data availability statement. This statement should provide the following information, where applicable:

- Accession codes, unique identifiers, or web links for publicly available datasets
- A description of any restrictions on data availability
- For clinical datasets or third party data, please ensure that the statement adheres to our policy

Atomic coordinates that support the findings of this study are available in the RCSB Protein Data Bank (PDB) under accession numbers 7LV2 [http://doi.org/10.2210/pdb7LV2/pdb], 7LTU [http://doi.org/10.2210/pdb7LTU/pdb] (form 1), 7LUX [http://doi.org/10.2210/pdb7LUX/pdb] (form 2), and 7LUZ [http://doi.org/10.2210/pdb7LUZ/pdb]. The amino acid sequences of the Nucleocapsid proteins of SARS-CoV-2 and SARS-CoV analyzed in this study are available on UniProtKB, accession numbers: P0DTC9 [https://covid-19.uniprot.org/uniprotkb/P0DTC9#Sequence], and P59595 [https://www.uniprot.org/uniprotkb/P59595/entry#sequences] respectively. Amino acid sequences of other coronavirus Nucleocapsid proteins were accessed from the European Nucleotide Archive [ENA; https://www.ebi.ac.uk/genomes/virus.html]. The raw EM images, light and fluorescence microscopy images and fiber diffraction source files generated in this study have been deposited in the Figshare respiratory at [https://figshare.com/projects/Low_Complexity_Domains_of_the_Nucleocapsid_Protein_of_SARS-CoV-2_Form_Amyloid_Fibrils/162391]. Data for all plots presented in this manuscript are provided with this paper in the Source Data file.

Human research participants

Policy information about studies involving human research participants and Sex and Gender in Research.

Reporting on sex and gender	N/A
Population characteristics	N/A
Recruitment	N/A
Ethics oversight	N/A

Note that full information on the approval of the study protocol must also be provided in the manuscript.

Field-specific reporting

Please select the one below that is the best fit for your research. If you are not sure, read the appropriate sections before making your selection.

☒ Life sciences ☐ Behavioural & social sciences ☐ Ecological, evolutionary & environmental sciences

For a reference copy of the document with all sections, see nature.com/documents/nr-reporting-summary-flat.pdf

Life sciences study design

All studies must disclose on these points even when the disclosure is negative.

Sample size	No statistical calculations were used to determine sample sizes. For Thioflavin-T (ThT) and turbidity kinetic experiments, and for phase separation (PS) experiments used for quantification (Figures 3 and 5), three biological repeats, each with technical triplicates were performed. For endpoint ThT experiment three replicates were tested.<br>Protein, RNA and G12 concentrations, as well as cell confluence and viral amounts were determined as following:<br>-ThT, Turbidity and PS experiments shown in Figures 1.d&e, 3, 5.b, and Supplementary Figures 2&6- Different protein and vRNA concentrations were screened around values used with this protein and other RNA segments in the literature [e.g. Perdikari, T. M. et al. EMBO J. 39, (2020); Savastano, A. et al. Nat. Commun. 11, 1–10 (2020)]. Plots and microscopy images presented in the figures were generated using optimal concentrations found in those initial screens.<br>-In vitro activity of G12 shown in figure 5b&c, and Supplementary Figures 6: Different NCAP:G12 molar ratios of 1:0.1 to 1:2 were screened in vitro. Molar ratios producing results ranging from minimal to maximal PS disturbing effects are shown in Figure 5. An effective PS disturbing ratio of 1:1 was chosen as representative ratio for Supplementary Figure 6.<br>In cell activity of G12 shown in figures 5d, and Supplementary Figures 7&8: Different G12 concentrations were screened in cells from low nM to the maximal concentration possible while keeping final DMSO concentration of 0.5% in all wells of the experiment. Cell confluence and amount of virus used for infection were determined based on our experience from previous studies [e.g. Garcia, G. et al. Cell Rep. 35, 108940 (2021)]. For Supplementary Figure 7 we used 15uM FITC-labeled G12 to show that this high concentration still allows the diffused distribution of G12 in the cells.<br>EM micrographs of fibrils and Fiber diffraction experiments shown in Figures 1, 2 and Supplementary Figure 2: Different protein concentrations of generally 10- 150 uM were tested for fibril formation under EM while maintaining the same protein:RNA molar ratio used for the ThT and turbidity experiments. For visualization of fibrils in EM we overall noticed that increased protein concentration is required compared to ThT experiments as elaborated in the manuscript.<br>Endpoint ThT experiment (Figure 1.g)- Protein concentration was similar to that showing fibril formation of this protein in EM.
Data exclusions	Figure 3d& Supplementary Figure 2f- the 600 nm absorbance was recorded every 5 minutes and the plots present data points for every 10 minutes of measurements for clarity of presentation and data markers. All data points collected complied with the trend of their respective curves without significant outliers.<br>Figure 5d& Supplementary Figure 8- due to technical limitations of quantitative immunofluorescence labeling and other steps of this assay, some tested G12 concentrations gave "noisier" results with rather high variability, however still allowing an overall clear dose-dependent response. In order to fit a linear regression model to our data nicely and estimate the IC50 we used the less noisy measurement points to generate the plot shown in Figure 5d. Nevertheless, we are showing all acquired data in Figure S8 for complete transparency.
Replication	Figures 1. d&e - the experiments were repeated at least three independent times with technical triplicates according to field standards showing similar results to ensure reproducibility. Representative curves are presented.<br>Figure 3b&c-the experiment was repeated three independent times with triplicates showing similar trend of transition from PS droplets to solid particles with some variation in particle size, circularity and ThS fluorescence that may result from batch to batch protein and RNA differences, pipettation inaccuracies and minor deviations in data acquisition times, especially at day1 of incubation. To account for this variability, data from all repeats (n=45 images per condition per time point) were combined for final data analysis that is shown in the figure. Images in Figure 3a were taken from another experiment identical to those analyzed in Figure 3b&c, only with one replicate and an additional imaging time point of 6 h of incubation.<br>Figure 1.g- The experiment was performed with three replicates.<br>Figure 3d- the experiment was repeated at least three independent times with triplicates showing similar results with the exception of the exact time in which the LCD+RNA curve reaches its minima. This variability may result from different assay preparation durations and measurement onset times, as well as from batch to batch protein and RNA variabilities. A representative curve is presented.<br>Figure 5c-the experiment was repeated three independent times with triplicates showing similar results. Representative boxplots are presented, with data analyzed from one biological repeat (n=15 images per condition).<br>Figure 5d& Supplementary Figure 8- we tested G12 in three biological repeats with triplicates using different G12 concentrations and viral titers (MOI between 0.02-0.1). G12 consistently showed a reduction of viral infectivity by ~40-60% at concentrations higher than ~10 uM. The complete dose response curve shown in Figure 5d& Supplementary Figure 8 was repeated once with triplicates.<br>Supplementary Figure 2. e&f- the experiments were repeated at least three independent times with triplicates according to field standards showing similar trends but with some variability in the delta between the NCAP only and NCAP+ RNA curves and noisiness of the data in panel e which may result from protein and RNA batch to batch variabilities and from low absorbance measurements overall. Representative curves are presented.<br>Figure 2.a- X-ray diffractions of LCD only and LCD + S2hp fibrils were each collected three times on different days, using different diffractometers and x-ray sources while showing similar results. Diffraction of LCD + non-specific RNA fibrils was collected twice from different regions of the same loop, showing similar results.<br>EM micrographs of LCD only fibrils were captured at least five independent times. LCD fibrils with the different vRNA segments were visualized by EM at least two independent times per vRNA type, once of which with different time points. NCAP with and without S2hp vRNA in PBS was imaged by EM from two independent samples. Other EM images were taken from a single sample.<br>Supplementary Figure 2.a- PS of NCAP with ThS was repeated twice (2nd repeat incubated for 3 days only) showing ThS partitioning into NCAP's PS droplets.<br>Supplementary Figure 6- FITC labeled G12 was tested on NCAP PS droplets in vitro once.<br>Supplementary Figure 7- Distribution of FITC labeled G12 in HEK293-ACE2 cells was tested two independent times with duplicated wells.
Randomization	In all experiments samples were grouped by molecular identity and treatment.
Blinding	Blinding was not relevant to this study because no subjective assessments of group or categories were involved, and the authors who performed each experiment also analyzed the data. There is no risk of bias.

Reporting for specific materials, systems and methods

We require information from authors about some types of materials, experimental systems and methods used in many studies. Here, indicate whether each material, system or method listed is relevant to your study. If you are not sure if a list item applies to your research, read the appropriate section before selecting a response.

Materials & experimental systems

n/a	Involved in the study
☐	☒ Antibodies
☐	☒ Eukaryotic cell lines
☒	☐ Palaeontology and archaeology
☒	☐ Animals and other organisms
☒	☐ Clinical data
☒	☐ Dual use research of concern

Methods

n/a	Involved in the study
☒	☐ ChIP-seq
☒	☐ Flow cytometry
☒	☐ MRI-based neuroimaging

Antibodies

Antibodies used

Two anti Spike-protein primary antibodies were used in our assays interchangeably, depending on their availability during the pandemic, while showing similar results:
1. BEI Resources (managed by ATCC), NIAID, NIH rabbit monoclonal Anti-SARS-Related Coronavirus 2 Spike Glycoprotein S1 Domain (produced in vitro), cat. no. NR-53788, clone no. 007 (purchased from SinoBiological cat. no. 40150-R007). Lot: HA14AP3001. Used at a 1:100 dilution ratio.
2. BEI Resources (managed by ATCC), NIAID, NIH: Mouse monoclonal Anti-SARS-CoV S Protein (Similar to 240C). Antibody Class: IgG2a. cat. no. NR-616. Lot: 102204. Used at a 1:300 dilution ratio.

Conjugated secondary antibodies:
3. Abcam, Goat Anti-Rabbit IgG H&L (Alexa Fluor® 555), cat. no. ab150078. Lot: GR302355-2. Used at 1:1000 dilution.
4. Abcam, Goat Anti-Mouse IgG H&L (Alexa Fluor® 555), cat. no. ab150114. Lot: GR299321-5. Used at 1:1000 dilution.

Validation

1. "ATCC , on behalf of BEI Resources, hereby represents and warrants that the material provided under this certificate has been subjected by the contractor to the tests and procedures specified and that the results described, along with any other data provided in this certificate, are true and accurate to the best of ATCC 's knowledge." Certificate could be downloaded here: https://www.beiresources.org/Catalog/BEIMonoclonalAntibodies/NR-53788.aspx. According to the manufacturer:" The biological activity of NR-53788 was measured by immunofluorescence staining (1:60) in ACE2-overexpressed 293T cells infected with 2019-nCoV-Spike pseudovirus."
2. "ATCC®, on behalf of BEI Resources, hereby represents and warrants that the material provided under this certificate has been subjected by the vendor to the tests and procedures specified and that the results described, along with any other data provided in this certificate, are true and accurate to the best of ATCC®'s knowledge." Certificate could be downloaded here: https://www.beiresources.org/Catalog/BEIMonoclonalAntibodies/NR-616.aspx. The specificity of the antibody was determined by reactivity to SARS-CoV S protein by ELISA and confirmed by Western blot analysis.
3. Antibody verified by manufacturer for use in immunofluorescence labeling under the "Abpromise guarantee". Information and citations could be found here: https://www.abcam.com/goat-rabbit-igg-hl-alexa-fluor-555-ab150078.html?productWallTab=ShowAll
4. Antibody verified by manufacturer for use in immunofluorescence labeling under the "Abpromise guarantee". Information and citations could be found here: https://www.abcam.com/goat-mouse-igg-hl-alexa-fluor-555-ab150114.html?productWallTab=ShowAll

Eukaryotic cell lines

Policy information about cell lines and Sex and Gender in Research

Cell line source(s)

HEK293-ACE2 cells stably over-expressing the human ACE2 receptor were established by the authors of this paper [Garcia, G. et al. Cell Rep. 35, 108940 (2021)]. The original HEK-293T cells were purchased from ATCC, cat. no. CRL3216. The sex of the original HEK293T cells is not provided by the vendor.

Authentication

STR profiling of the original HEK293T cells (ATCC, CRL3216): CSF1PO: 11,12; D13S317: 12,14; D16S539: 9,13; D5S818: 8,9; D7S820: 11;TH01: 7, 9.3; TPOX: 11.vWA: 16,19; Amelogenin: X.

Mycoplasma contamination

HEK293-ACE2 cells used in this study were confirmed negative for mycoplasma by PCR using a Universal Mycoplasma Detection Kit (ATCC cat. no. 30-1012K).

Commonly misidentified lines
(See ICLAC register)

No misidentified cell lines were used in this study.
